# Supplementary material for: Hyperglycemia and Hypoglycemia Are Associated with In-Hospital Mortality among Patients with Coronavirus Disease 2019 Supported with Extracorporeal Membrane Oxygenation
Source: J Clin Med. 2022 Aug 30;11(17):5106. doi: 10.3390/jcm11175106 (PMC9457381; doi:10.3390/jcm11175106)

Supplementary Table S1. Missing data

Variable	Number of missing	Variable	Number of missing
Male sex	0	BT	7
Age	0	WBC	0
Hight	0	BUN	2
Weight	0	Creatinine	12
BMI	0	AST	4
Hypertension	0	ALT	4
History of diabetes	0	Initial glucose	16
Chronic renal disease	0	Glucose before ventilator	53
Cerebral disease	0	Glucose before ECMO	55
Cancer	4	Max glucose	53
Heart failure	0	Min glucose	56
Ischemic heart disease	0	SOFA	53
Dyslipidaemia	0	PaO_2/FiO_2 ratio before ECMO	16
Arrhythmia	0	Ventilator to ECMO >7 days	4
COPD	0	Cardiac arrest before ECMO start	8
Smoking	0	Inotropic use before ECMO	12
Initial systolic BP	6	Steroid use	14
Initial diastolic BP	6	RRT before ECMO	9
HR	6	Mortality	0

BP, blood pressure; HR, heart rate; BT, body temperature; WBC, white blood cell; BUN, blood urea nitrogen; AST, aspartate aminotransferase; ALT, alanine aminotransferase; ECMO, extracorporeal membrane oxygenation; SOFA, sequential organ failure assessment; PaO_2/FiO_2, partial pressure of arterial oxygen over fractional inspired oxygen. RRT, renal replacement therapy

Supplementary Table S2. Patients 'characteristics

**Variable**	**Survivors (n = 85)**	**Nonsurvivors (n = 110)**	**p-value**
Male sex	60 (70.6)	76 (69.1)	0.47
Age, years	60 (50-60.5)	64 (56-69)	0.002
Hight, cm	166.32 (9.17)	165.43 (7.35)	0.45
Weight, kg	73 (65-86)	69 (62-78)	0.01
BMI, kg/m^2	26.4 (24.55-30.95)	25.25 (23.3-27.45)	0.06
Hypertension	47 (55.3)	56 (50.9)	0.28
Diabetes	21 (24.7)	46 (39.1)	0.02
Chronic renal disease	4 (4.7)	5 (4.5)	0.70
Cerebral disease	4 (4.7)	8 (7.3)	0.29
Cancer	0	7 (6.4)	0.04
Heart failure	2 (2.4)	2 (1.8)	0.68
Ischemic heart disease	1 (1.2)	5 (4.5)	0.22
Dyslipidaemia	15 (17.6)	15 (13.6)	0.44
Arrhythmia	1 (1.2)	1 (0.9)	0.70
COPD	0	1 (0.9)	0.63
Smoking			0.01

Current smoker	9 (10.6)	4 (3.6)
Ex-smoker	11 (12.9)	12 (10.9)
Never smoker	58 (68.2)	67 (60.9)

Values are presented as mean (SD) or median (IQR) or n (%).

BMI, body mass index; COPD, chronic obstructive pulmonary disease

Supplementary Table S3. Data of initial vital signs and laboratory test

Variable	Survivors (n = 85)	Nonsurvivors (n = 110)	*p*-value
Initial systolic BP, mmHg	133 (117-149.75)	127 (112-149)	0.42
Initial diastolic BP, mmHg	82.37 (15.97)	75.30 (16.16)	<0.01
HR, /min	88.5 (76-109.5)	91 (76.5-102)	0.83
BT, ℃	37.10 (36.7-37.5)	37 (36.5-37.6)	0.61
WBC, $10^3/mm^3$	8.4 (5.44-12.04)	7.45 (5.36-12.02)	0.59
BUN, mg/dL	18.8 (14.65-23.9)	20.5 (14.88-30.11)	0.09
Creatinine, mg/dL	0.82 (0.66-1.16)	0.87 (0.65-1.35)	0.63
AST, U/L	50 (34-83)	53 (40.9-88.75)	0.26
ALT, U/L	35 (22-55.5)	37 (24-65)	0.49
Initial glucose, mg/dL	148 (125-198)	154 (115-213)	0.99
Glucose before ventilator, mg/dL	156 (130.5-186.25)	159.5 (121-247.75)	0.64
Glucose before ECMO, mg/dL	172.5 (138-229.5)	202.5 (159-278)	0.01
Max glucose, mg/dL	320.5 (241.25-405.25)	339 (275.25-421.75)	0.33
Min glucose, mg/dL	103 (87.5-121.5)	85 (59-108)	<0.01
SOFA	7.5 (6-10)	9 (7-12)	0.02
PaO_2/FiO_2 ratio before ECMO, mmHg	76.95 (59.93-100.68)	73.09 (59.85-97.06)	0.55
Ventilator to ECMO >7 days	15(17.9)	30(28.0)	0.10
Cardiac arrest before ECMO start	5 (6.0)	10 (9.7)	0.35
Inotropic use before ECMO	44 (53.7)	61 (60.4)	0.36
Steroid use	73 (98.6)	104 (97.2)	0.51
RRT before ECMO	4 (4.8)	15 (14.7)	0.03

Values are presented as mean (SD) or median (IQR) or n (%).

BP, blood pressure; HR, heart rate; BT, body temperature; WBC, white blood cell; BUN, blood urea nitrogen; AST, aspartate aminotransferase; ALT, alanine aminotransferase; ECMO, extracorporeal membrane oxygenation; SOFA, sequential organ failure assessment; PaO_2/FiO_2, partial pressure of arterial oxygen over fractional inspired oxygen. RRT, renal replacement therapy

Supplementary Table S4. Cox regression analysis for mortality after ECMO support in COVID-19

	Model 1		Model 2		Model 3		Model 4	
Variable	HR (95% CI)	*p*-value	HR (95% CI)	*p*-value	HR (95% CI)	*p*-value	HR (95% CI)	*p*-value
Age, years								
< 50	Reference		Reference		Reference		Reference	
50–59	1.13 (0.45–2.83)	0.80	1.06 (0.41–2.76)	0.91	0.95 (0.40–2.23)	0.90	1.20 (0.59–2.42)	0.62
60–69	1.79 (0.84–3.83)	0.13	1.93 (0.86–4.36)	0.11	1.60 (0.80–3.22)	0.19	1.69 (0.93–3.06)	0.09
≥70	2.25 (0.98–5.18)	0.06	2.73 (1.11–6.70)	0.03	2.07 (0.91–4.67)	0.08	1.81 (0.89–3.70)	0.10
BMI, kg/m^2								
<18.5	9.78 (2.10–45.66)	<0.01	9.48 (2.07–43.39)	<0.01	13.80 (2.90–65.62)	<0.01	11.35 (2.56–50.40)	<0.01
18.5–24.9	Reference		Reference		Reference		Reference	
≥25	0.88 (0.54–1.43)	0.59	0.78 (0.48–1.26)	0.31	0.85 (0.52–1.38)	0.51	0.97 (0.63–1.48)	0.87
RRT before ECMO	2.19 (1.04–4.59)	0.04	2.25 (1.15–4.41)	0.02	3.09 (1.62–6.38)	<0.01	2.49 (1.39–4.48)	<0.01
Glucose before ventilator >200 mg/dL	1.47 (0.89–2.43)	0.14	-	-	-	-	-	-
Glucose before ECMO >200 mg/dL	-	-	1.93 (1.19–3.13)	<0.01	-	-	-	-
Minimal glucose<70 mg/dL	-	-	-	-	3.61 (2.15–6.05)	<0.01	-	-
Diabetes history	-	-	-	-	-	-	1.32 (0.87–2.00)	0.19

ECMO, extracorporeal membrane oxygenation; RRT, renal replacement therapy; DM, diabetes mellitus; BMI, body mass index; HR, hazard ratio; CI, confidence interval

Supplementary Figure S1. Survival curves

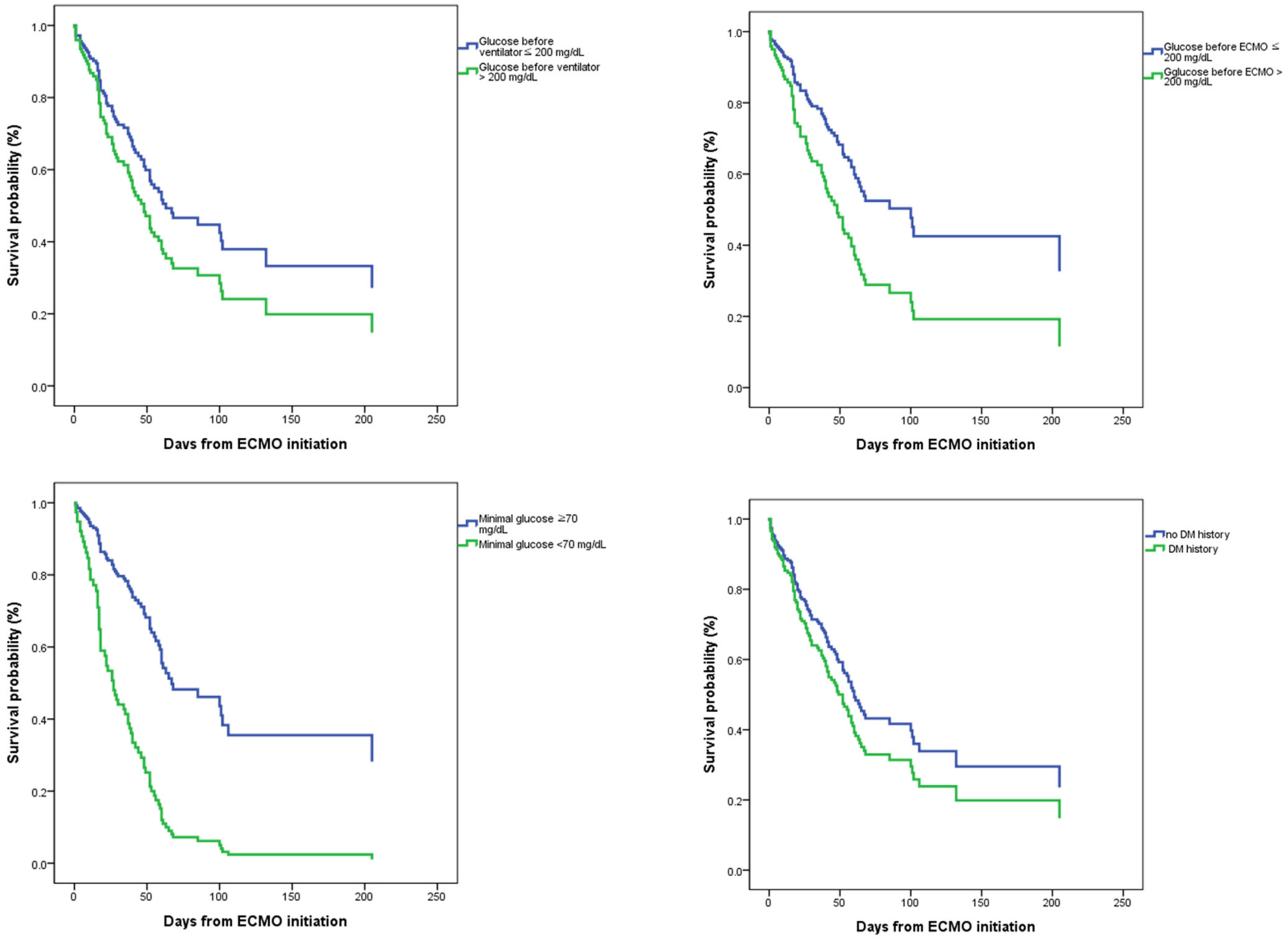

Supplementary Figure S2. Survival curves

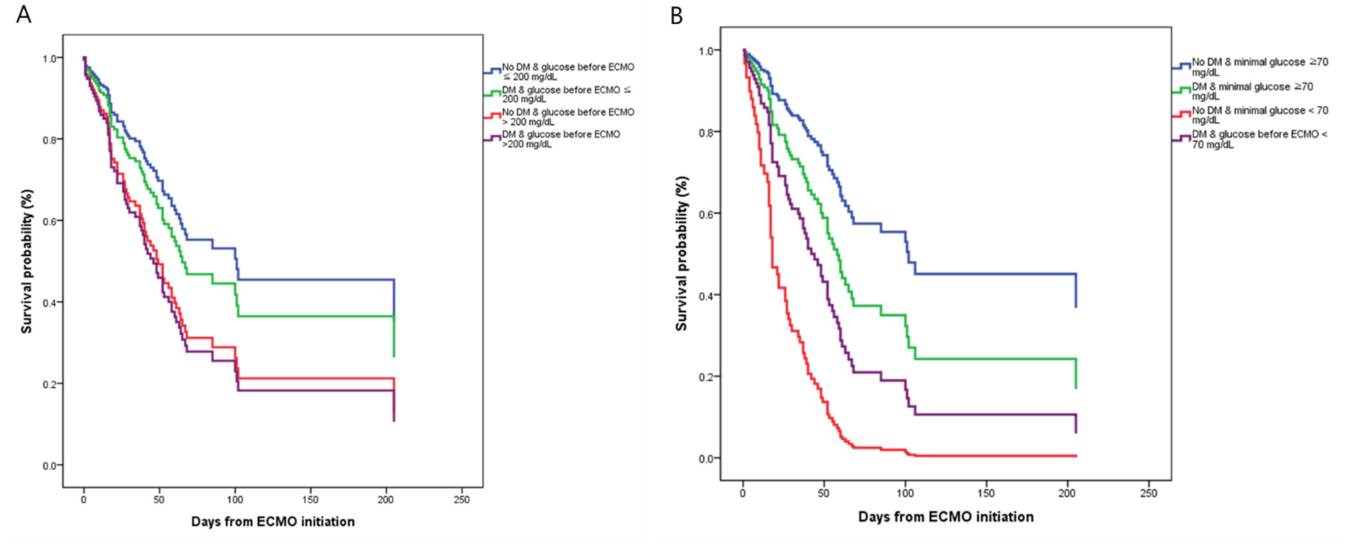

Supplement: Supplementary file 1 [file jcm-11-05106-s001.zip › jcm-1847734-supplementary.pdf]
